# Thriving despite aging: how attitudes toward own aging empower thriving at work

**DOI:** 10.3389/fpsyg.2026.1959203

**Published:** 2026-09-25

**Authors:** Huan Yang, Sansan Zeng, Tong Li

**Affiliations:** 1 School of Economics and Management, Shihezi University, Shihezi, China; 2 School of Business, Sun Yat-sen University, Guangzhou, China

**Keywords:** attitudes toward own aging, conservation of resources theory, gender, perceived occupational stress, thriving at work

## Abstract

**Background:**

Against the backdrop of ongoing population aging, how to activate the work vitality of older employees aged 45 and above has become an urgent challenge for organizational human resource management.

**Method:**

Drawing upon Conservation of Resources (COR) theory, this study constructs a moderated mediation model and employs a mixed-method research design combining a scenario-based experiment (*N* = 178) and a questionnaire survey (*N* = 225) to systematically investigate the underlying mechanisms and boundary conditions through which attitudes toward own aging empower thriving at work among older employees.

**Results:**

Positive attitudes toward own aging predict thriving at work; perceived occupational stress mediates the relationship between attitudes toward own aging and thriving at work; gender moderates the indirect effect of perceived occupational stress. Specifically, for female employees, the negative effect of positive attitudes toward own aging on perceived occupational stress is stronger, and consequently, the facilitating effect on individual thriving at work is also more pronounced.

**Conclusion:**

These findings not only elucidate the underlying mechanisms and boundary conditions by which attitudes toward own aging influence individual thriving at work, but also provide theoretical and practical implications for effectively enhancing the thriving level of older employees.

## 1 Introduction

Against the macroeconomic backdrop of the ongoing development of the digital economy and the deepening population aging, organizational phenomena such as the “optimization of older employees” and external pressures from an increasingly competitive job market have led to growing concerns among employee concerns aging. These concerns not only place significant psychological pressure on older employees but also dampen their enthusiasm for work and motivation to learn. Against this backdrop, transforming the “burden of age” for older employees into an “asset” has become an urgent practical issue that demands a response. Focusing on the empowerment and professional growth of older employees is not only a practical necessity for alleviating the challenges posed by an aging society but also a strategic move to proactively align with delayed-retirement policies.

Enterprises typically designate age 45 as the demarcation point between younger and older employees, and allocate work resources and opportunities based on age, thereby intensifying the age-related stereotypes faced by employees aged 45 and above (Cui et al., 2025; Nagy et al., 2019a,b). However, differences among employees lie not only in chronological age but also in their attitudes toward aging. Attitudes toward own aging are distinct from constructs such as optimism and self-efficacy; they refer specifically to individuals’ self-evaluation of their own aging process, rather than general outcome expectancies or beliefs about personal capabilities (Diehl et al., 2014). Attitudes toward own aging also differ from subjective age, which is a comparative judgment, whereas Attitudes toward own aging represent a cognitive-evaluative judgment (Spuling et al., 2020). Meta-analytic evidence has confirmed that attitudes toward own aging are distinguishable from both chronological age and subjective age, and demonstrate incremental validity over and above these constructs (Yang et al., 2024). These findings underscore the theoretical uniqueness of attitudes toward own aging as a meaningful psychological construct. Existing research indicates that positive attitudes toward own aging are beneficial to physical health (Wettstein et al., 2020), improve cognitive functioning (Yang et al., 2024), and sustain positive emotions (Diener et al., 2006). Thus, older employees’ attitudes toward own aging may help stimulate their work vitality and capacity for continuous learning. Nevertheless, research on attitudes toward own aging has predominantly been situated within gerontology and geriatric medicine, with limited consideration in organizational management research and scant attention to cognitive–psychological processes in the workplace (Klusmann et al., 2012; Wurm et al., 2010).

Grounded in Conservation of Resources (COR) theory, this study explores how attitudes toward own aging empower older employees to exhibit higher levels of thriving at work. COR theory posits that individuals are inclined to preserve, maintain, and acquire resources that they value. Individuals with abundant resources are more capable of retaining and acquiring additional resources and are more willing to invest resources, thereby generating resource gain spirals (Hobfoll, 2001). Employees with positive attitudes toward own aging pay greater attention to the positive aspects of aging, view age growth as an advantage in resource accumulation (Blawert and Wurm, 2021), and thus invest in and acquire resources more proactively, ultimately exhibiting higher levels of vitality and learning capacity. Furthermore, when individuals face actual or potential resource loss, they experience negative psychological reactions (Hobfoll, 2001). Older employees with negative attitudes toward own aging tend to worry about resource depletion, thereby heightening their perceived stress. By contrast, those with positive attitudes toward own aging are more accepting of the negative consequences of aging, perceive less resource loss, and are more willing to invest resources to manage stress (Hobfoll et al., 1990; Yang et al., 2024). Accordingly, this study introduces perceived occupational stress as a key variable in the research model examining the influence of attitudes toward own aging on individual thriving at work, with the aim of exploring its mediating role. Meanwhile, men and women differ substantially in workplace resources and age-related evaluative standards (Putrevu, 2001). Most existing research has treated the labor market as a homogeneous whole, with limited micro-level examination of subgroup differences, particularly between men and women. Therefore, this study investigates gender differences in the conversion of personal resources into work resources.

In summary, drawing upon COR theory, this study examines the impact of attitudes toward own aging on older employees’ individual thriving at work, further explores the mediating role of perceived occupational stress, and incorporates gender as a critical contextual moderator. On the one hand, this study extends the literature on the consequences of attitudes toward own aging in the workplace, responding to the urgent scholarly call for greater attention to older employees. On the other hand, it encourages organizational managers to shift from passively responding to population aging to proactively investing in older human resources, leveraging older employees’ “positive mindsets” to develop their capabilities, thereby providing a win–win pathway for both successful aging and sustained organizational development.

## 2 Literature review and hypotheses

### 2.1 Attitudes toward own aging and older employees’ thriving at work

Positive attitudes toward own aging represent a critical psychological resource for older employees confronting age-related changes (Yang et al., 2024). Conservation of Resources (COR) theory posits that individuals strive to protect and acquire valued resources, and that both resource gain and loss are important determinants of stress (Hobfoll, 2001). Within this framework, attitudes toward own aging represent a cognitive resource that shapes how older employees interpret their aging experience. When aging is viewed as a period of continued growth and resource accumulation, employees become more inclined to adopt proactive resource investment behaviors. Thriving at work refers to the psychological state in which individuals simultaneously experience learning and vitality (Spreitzer et al., 2005), which involves proactively investing resources and time into work activities. The learning dimension reflects employees’ proactive acquisition of new knowledge, skills, and experiences, emphasizing the realization of potential and personal growth; the vitality dimension reflects employees’ energetic and enthusiastic engagement in work, emphasizing positive affective experiences (Shrestha and Tamrakar, 2026). Drawing on COR theory, this study proposes that positive aging attitudes promote thriving at work via two complementary pathways.

First, they help preserve the health-related resources that sustain work vitality. As COR theory posits, resources do not exist in isolation but in resource caravans, where one resource enables the acquisition of others (Hobfoll, 2001). Positive aging attitudes act as a key catalyst for initiating these resource caravans. Prior research has shown that positive attitudes toward own aging improve health outcomes and benefit physical health (Wettstein et al., 2023). For example, such attitudes are associated with reduced disease incidence (Ayalon and Cohn-Schwartz, 2022) and lower mortality rates (Wang et al., 2023). They are also associated with better physician-rated health status (Wettstein et al., 2020). In addition, sound cognitive functioning supports older employees’ learning efficiency and problem-solving abilities, which are conducive to thriving. Positive attitudes toward own aging mitigate cognitive decline (Yang et al., 2024), which is critical for learning new knowledge and technologies and for handling work tasks. Conversely, negative attitudes toward own aging increase the risk of future illness (Siebert et al., 2018). Older employees in good health possess greater energy, are better able to maintain vitality at work, and are less likely to experience work interruptions due to sick leave, thereby enabling the continuous acquisition of new knowledge and skills. This combined preservation of physical and cognitive resources creates the foundational conditions for sustaining and reinforcing older employees’ vitality and learning capacity.

Second, positive aging attitudes activate positive emotions, which in turn motivate employees to proactively invest resources, thereby strengthening their motivational orientation toward learning. COR theory emphasizes that individuals with greater resource reservoirs are more willing to invest resources, whereas resource-depleted individuals tend to adopt defensive, resource-conservation strategies that constrain proactive development (Hobfoll, 2001). Individuals with positive attitudes toward own aging focus more on positive feelings and experiences, exhibit more upbeat emotions, report higher life satisfaction (Brothers et al., 2015), feel happier and more relaxed (Diener et al., 2006), and are more confident (Klusmann et al., 2019). This positive emotional state signals to the individual that sufficient resources are available, encouraging proactive resource investment—including the pursuit of learning opportunities and engagement in work. Consistent with this logic, Socioemotional Selectivity Theory (SST) suggests that employees who hold positive attitudes are more likely to experience positive emotions and maintain an open future time perspective, thereby becoming more willing to invest effort for future gains (Carstensen, 2021). Positive and pleasurable experiences encourage older employees to believe they have sufficient time and resources to overcome challenges and achieve desired outcomes (Yang et al., 2024; Ye et al., 2026).

Taken together, these two pathways, namely health resource preservation and motivational resource investment, explain why older employees with positive aging attitudes tend to perform better at work. By preserving the physical and cognitive resources that sustain vitality and activating the motivational resources that drive learning, positive aging attitudes create the fundamental conditions for both the learning and vitality dimensions of thriving at work. Based on this reasoning, we propose Hypothesis 1:

*Hypothesis 1 (H1)*: Attitudes toward own aging positively influence older employees’ thriving at work.

### 2.2 The mediating role of perceived occupational stress

According to COR theory, actual or anticipated loss of valued resources constitutes the primary source of stress (Hobfoll, 2001). This premise is especially pertinent to older employees, who confront multiple resource threats as they age, such as career termination, physical decline, and diminished energy (Cui et al., 2025). However, whether these objective changes translate into subjectively experienced stress depends critically on how employees interpret the meaning of own aging. COR theory uniquely explains this differentiation: stress arises not merely from objective resource loss, but from the appraisal of resource loss (Hobfoll, 2001). Older employees with negative attitudes toward own aging amplify this appraisal process by directing attention to the negative aspects of age-related changes, thereby intensifying the perception of resource depletion and triggering stress responses in the workplace (Hobfoll, 2002). Perceived occupational stress refers to employees’ cognitive appraisals and evaluations of potential threats in the work environment, giving rise to a series of physiological, psychological, and behavioral reactions when coping capacities are insufficient (Veltri et al., 2026; Zhang et al., 2026). Conversely, older employees with positive attitudes toward own aging focus more on the positive aspects of aging, perceiving increases in resources such as experience, wisdom, and status. They adopt a more accepting stance toward the negative consequences of aging, thereby attenuating sensitivity to resource loss and reducing the allocation of attention, emotional, and other resources to self-regulation, which in turn curbs resource depletion (Kunze et al., 2015; Lazazzara and Za, 2019).

Moreover, positive aging attitudes not only attenuate the perception of resource loss but also activate resource gain spirals that further buffer stress (Yang et al., 2024). COR theory also suggests that resource-rich individuals are better able to cope with stress (Hobfoll et al., 1990). Older employees who possess positive attitudes toward own aging evaluate their personal resources more optimistically and demonstrate a greater propensity for proactive resource-investment strategies, including the pursuit of novel learning opportunities and the maintenance of work engagement. These strategies serve a dual function: they alleviate immediate occupational stress while simultaneously generating resource gains for the future. The Selection, Optimization, and Compensation (SOC) framework further corroborates this reasoning, suggesting that employees with positive aging attitudes are more predisposed to employ selection, optimization, and compensation strategies to reallocate their resources in pursuit of successful aging (Zacher et al., 2015). Conversely, employees with negative aging attitudes tend to eschew SOC strategies, thereby intensifying the occupational stress associated with resource loss.

Moreover, lower levels of perceived occupational stress are conducive to enhancing employees’ thriving at work. Existing research indicates that work stress depletes energy, reduces satisfaction, and impairs performance (De Gieter et al., 2018). Reduced perceived occupational stress can diminish psychological exhaustion among older employees, sustain their vitality at work, and promote proactive learning (Chen et al., 2024; Li et al., 2025). As older employees with positive attitudes toward own aging invest resources to cope with stress, these investments also generate future resource gains, fostering personal growth (Hoppmann et al., 2017) and ultimately enhancing thriving at work. Hence, we propose Hypothesis 2:

*Hypothesis 2 (H2)*: Perceived occupational stress mediates the relationship between attitudes toward own aging and older employees’ individual thriving at work.

### 2.3 The moderating role of gender

COR theory suggests that individuals’ occupational stress stems from the continual depletion of workplace resources (Hobfoll, 2001). Positive attitudes toward own aging, as a protective psychological resource, can compensate for resource loss triggered by age growth. However, men and women have long faced differentiated workplace resource constraints and age-evaluation standards, with distinct role expectations and behavioral norms (Adriaans et al., 2025; Pandey et al., 2021; Ridgeway, 2009). When confronted with the same work environment, men and women differ across physiological, psychological, cognitive, and behavioral dimensions (Man et al., 2026; Putrevu, 2001). Accordingly, the efficacy of psychological resources in alleviating stress may vary by gender. This study thus examines how inherent gender shapes the resource flow process.

According to the stereotype content model, social judgments are organized along two fundamental dimensions: warmth and competence (Cundiff et al., 2026; Fiske et al., 2002). Prior research demonstrates that middle-aged professional women are perceived as agentic (competent) yet experience a decline in warmth from young adulthood to middle age, rendering them incongruent with the communal stereotypes prescribed for women (Chatman et al., 2022). By contrast, middle-aged men do not experience the same warmth-related decline, as their agency aligns more closely with masculine stereotypes (Chatman et al., 2022). Socioemotional Selectivity Theory further suggests that individuals tend to prioritize emotionally meaningful social relationships and pursue positive emotional experiences over instrumental goals (Nikitin et al., 2024). Gender differences in the use of positive psychological resources (Chui and Diehl, 2023). Women’s perceived occupational stress is shaped by factors such as interpersonal relationship stability, resource adequacy, and recognition of self-worth. Thus, for older female employees, positive attitudes toward own aging—as a cognitive resource—enhance their attention to and pursuit of positive emotional experiences, driving them to proactively build and maintain high-quality social connections. This process effectively compensates for perceived warmth deficits in the workplace and buffers the psychological distress arising from stereotype incongruity. For older male employees, whose age-related stereotypes center primarily on competence devaluation (e.g., declines in physical functioning and ability), the buffering effect of positive aging attitudes is comparatively limited, as it addresses only a subset of the stereotype content relevant to men’s aging concerns.

Furthermore, this gendered pattern of stereotype content is compounded by differential exposure to age and gender discrimination. Research indicates that workplace ageism is strongly associated with negative self-perceptions of aging, and this effect is more pronounced among men (Takeuchi and Katagiri, 2024). However, women are more likely than men to experience age discrimination in the workplace and are more likely to be restricted in their career development (Hideg and Ferris, 2016; Jones et al., 2014). These findings suggest that the same positive aging attitude may have different “conversion efficiencies” in alleviating occupational stress. For women who experience multiple forms of discrimination (Sugisawa et al., 2026), positive aging attitudes help them cope with both the competence and warmth dimensions of stereotype threat, making them a more potent resource for countering multiple stereotype threats and thereby more effectively alleviating women’s occupational stress. For older male employees, whose age-related concerns are more directly tied to objective workplace performance evaluations, the buffering effect of positive aging attitudes is comparatively limited. Based on the above, we propose Hypothesis 3:

*Hypothesis 3 (H3)*: Gender moderates the relationship between positive attitudes toward own aging and perceived occupational stress, such that the negative relationship is stronger among older female employees than among older male employees.

Building on Hypothesis 3, compared with men, older female employees are more adept at translating the cognitive resources embedded in positive attitudes toward own aging into stress-reduction efficacy (Brody, 2018). Therefore, when women hold positive attitudes toward own aging, they are better able to resist discrimination, balance work and life, and achieve greater reductions in perceived occupational stress. By contrast, older male employees face less age-related pressure, and thus the reduction in perceived occupational stress associated with positive attitudes toward own aging is less pronounced. Accordingly, we further propose a moderated mediation model in which the indirect effect of attitudes toward own aging on thriving at work via perceived occupational stress is moderated by gender. Specifically, among older female employees, positive attitudes toward own aging are associated with greater reductions in perceived occupational stress and consequently higher levels of thriving at work. Thus, we propose Hypothesis 4:

*Hypothesis 4 (H4)*: Gender moderates the indirect effect of perceived occupational stress in the relationship between attitudes toward own aging and thriving at work, such that the indirect effect is stronger for older female employees than for older male employees.

The theoretical model of this study is depicted in Figure 1.

**Figure 1:**
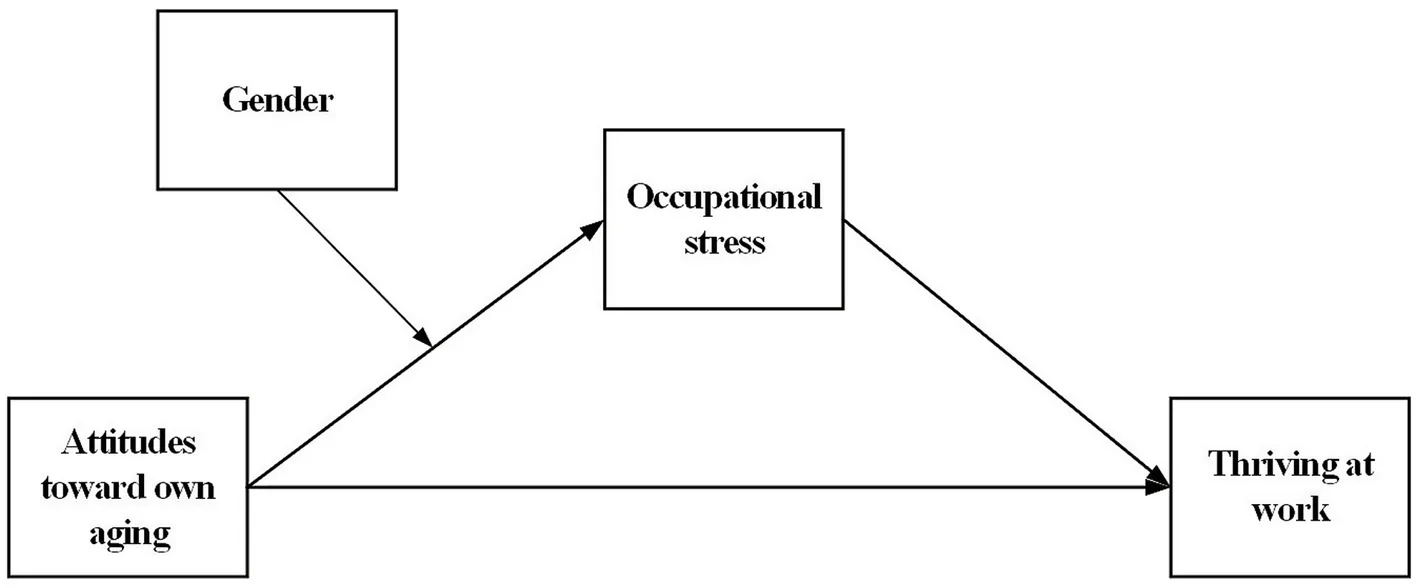
Theoretical model.

## 3 Study 1: experimental study

### 3.1 Participants

A total of 180 older employees with work experience were recruited online for a between-subjects experiment, with targeted recruitment of employees aged 45 and above. After excluding participants who failed to correctly identify the experimental scenario, 178 valid responses were retained, including 90 in the positive attitudes toward own aging condition and 88 in the negative attitudes condition. According to G*Power 3.1 power analysis, with a sample size of 178, α = 0.05, and *f* = 0.25, the statistical power was 0.91 (> 0.8), indicating adequate sample size (Maxwell, 2004). Men accounted for 40.4% of the sample, and women accounted for 59.6%. Age ranged from 45 to 79 years (*M* = 53, *SD* = 5.95). Tenure ranged from 3 to 50 years (*M* = 28.16, *SD* = 6.63).

### 3.2 Manipulation and measures

Drawing on Lawton’s Philadelphia Geriatric Center Morale Scale (Lawton, 1975), we designed the experimental materials for Study 1. Participants first read the instructions and were asked to imagine themselves in a mid-career workplace role, after which they were randomly assigned to either a positive or a negative attitude toward own aging condition. The experimental materials were as follows:

Positive attitudes toward own aging: “*As you age, you continue to acquire various workplace resources. Aging makes your professional judgment more insightful, enriches your experience, and steadily increases your opportunities of development. The workplace advantages brought by aging are irreplaceable, and your job value continues to rise. For you, aging only enriches your resources and solidifies your competitive advantage*.”

Negative attitudes toward own aging: “*As you age, you continue to experience various workplace resource losses. Aging reduces your learning efficiency, gradually depletes your energy, and narrows your promotion opportunities. The workplace disadvantages brought by aging are difficult to compensate for, and your job value continues to depreciate. For you, aging only depletes your resources, making it difficult to maintain your competitive advantage.*”

After reading the materials, participants completed the following measures, all rated on a 5-point Likert scale (1 = strongly disagree, 5 = strongly agree).

Manipulation check: Using the Philadelphia Geriatric Center Morale Scale (Lawton, 1975), a 5-item scale with higher scores indicating more positive attitudes toward own aging. Sample item: “Things keep getting better as I get older.” Cronbach’s *α* = 0.925.

Perceived occupational stress: Using the scale developed by Pearlin and Schooler (1978), consisting of 7 items, with higher scores indicating greater perceived occupational stress. Sample item: “When you think about your daily work, how do you feel—annoyed or frustrated?” Cronbach’s *α* = 0.893.

Thriving at work: Using the scale developed by Porath et al. (2012), consisting of 10 items, with 5 items for learning and 5 for vitality. Sample learning item: “At work, I find myself learning often.” Sample vitality item: “At work, I feel alive and vital.” Cronbach’s *α* = 0.855.

Participants then reported their gender, age, and tenure, and received a cash reward of 1.5 RMB upon completion.

### 3.3 Results

#### 3.3.1 Manipulation check

An independent-samples t-test revealed that the manipulation of attitudes toward own aging was successful. The positive condition (*M* = 4.14, *SD* = 0.53) and negative condition (*M* = 2.33, *SD* = 1.06) differed significantly, *t*(176) = 14.41, *p* < 0.001, Cohen’s *d* = 2.15.

#### 3.3.2 Hypothesis testing

Thriving at work was significantly higher in the positive condition (*M* = 4.32, *SD* = 0.27) than in the negative condition (*M* = 3.01, S*D* = 1.21), *t*(176) = 10.23, *p* < 0.001, Cohen’s *d* = 1.49, supporting H1. Similarly, perceived occupational stress was significantly lower in the positive condition (*M* = 1.71, S*D* = 0.46) than in the negative condition (*M* = 3.32, *SD* = 1.17), *t*(176) = −12.16, *p* < 0.001, Cohen’s *d* = −1.81.

To test the mediating role of perceived occupational stress, we employed PROCESS 4.1 Model 4 with 5,000 bootstrap resamples. The indirect effect of attitudes toward own aging on thriving at work via perceived occupational stress was 0.49 (*SE* = 0.06), with a bias-corrected 95% confidence interval of [0.37, 0.62], that excludes zero, supporting H2. Using SPSS 27, we conducted ANOVA to test the moderating role of gender. The interaction between attitudes toward own aging and gender on perceived occupational stress was significant, *F*(1,174) = 4.79, *p* = 0.03, *η*^2^ = 0.027. Simple effects analysis (as shown in Figure 2) showed that among women, perceived occupational stress was significantly lower in the positive condition (*M* = 1.66, *SD* = 0.12) than in the negative condition (*M* = 3.52, *SD* = 0.12). Among men, perceived occupational stress was also lower in the positive condition (*M* = 1.77, *SD* = 0.14) than in the negative condition (*M* = 3.04, *SD* = 0.14), but the difference was smaller. These results provide preliminary support for H3.

**Figure 2:**
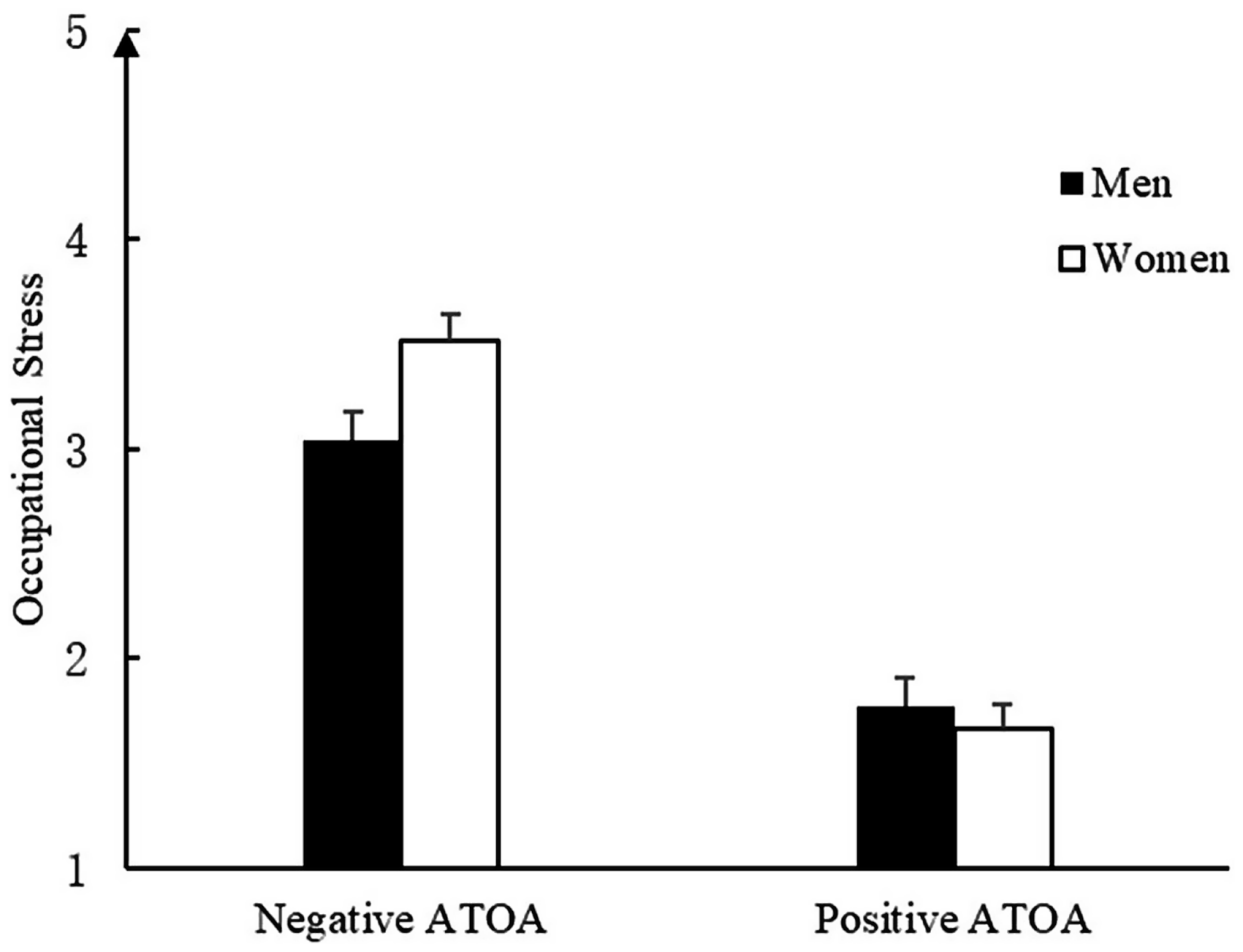
Moderating effect of gender (Study 1).

To test H4, we employed PROCESS 4.1 Model 7, including attitudes toward own aging (independent variable), perceived occupational stress (mediator), thriving at work (dependent variable), and gender (moderator; 1 = male, 0 = female). As shown in Table 1, for men, the indirect effect of attitudes toward own aging on thriving at work via perceived occupational stress was significant, *β* = 0.39, *SE* = 0.08, 95% CI [0.24, 0.55]. For women, this indirect effect was also significant, *β* = 0.57, *SE* = 0.07, 95% CI [0.43, 0.72]. The difference between genders was significant, *β*_high–low_ = −0.18, *SE* = 0.09, 95% CI [−0.36, −0.01]. Thus, H4 was supported. These findings indicate that the transmission of positive attitudes toward own aging to thriving at work via perceived occupational stress differs by gender, with a stronger stress-buffering effect among women, and consequently a stronger promoting effect on thriving.

**Table 1:** Mediating effects of perceived occupational stress by gender (Study 1).

Moderator	Effect	*SE*	95%CI	
			Lower	Upper
Female	0.57	0.07	0.43	0.72
Male	0.39	0.08	0.24	0.55
Difference	−0.18	0.09	−0.36	−0.01

## 4 Study 2: a three-wave time-lagged survey design

### 4.1 Sample and data source

The survey targeted older employees aged 45 and above. A three-wave time-lagged survey design was employed. At Time 1, 300 questionnaires were distributed to collect demographic information, including attitudes toward own aging, gender, and chronological age. The Time 2 survey was administered 1 week after Time 1; due to participant attrition, 242 questionnaires were returned, primarily measuring the mediator, perceived occupational stress. Time 3 data collection focused on thriving at work. After excluding incomplete responses and further dropouts, 225 valid questionnaires were ultimately retained, representing an effective response rate of 75%. Among the final sample, 53.8% were male and 46.2% female; the majority held a bachelor’s degree (62.2%); work tenure was primarily concentrated in the 20–29 years range (72.4%), with an average tenure of 31 years; 80.9% were junior or middle-level managers; and age ranged from 45 to 58 years, with a mean age of 48 years.

### 4.2 Measures

Consistent with Study 1, all measures were adopted from established scales and followed a standard translation-back-translation procedure to ensure accuracy. All items were rated on a 5-point Likert scale (1 = strongly disagree, 5 = strongly agree). Reverse-scored items were recoded to ensure directional consistency. Cronbach’s *α* values for attitudes toward own aging, perceived occupational stress, and thriving at work were 0.925, 0.893, and 0.855, respectively. Gender was coded as a categorical variable (male = 1, female = 0).

### 4.3 Results

#### 4.3.1 Common method variance

Harman’s single-factor test was conducted using SPSS 27.0. The first principal component accounted for 41.455% of the total variance, below the 50% threshold, indicating no serious common method bias. Additionally, the variance inflation factors (VIFs) for perceived occupational stress and attitudes toward own aging were 1.67, below the cutoff of 5, suggesting no severe multicollinearity.

#### 4.3.2 Confirmatory factor analysis

Convergent validity was assessed using average variance extracted (AVE). The AVE values for attitudes toward own aging, perceived occupational stress, and thriving at work were 0.716, 0.55, and 0.374, respectively, meeting the acceptable criteria (> 0.5 for the first two and > 0.36 for the latter). Confirmatory factor analysis (CFA) using Amos 26.0 tested the discriminant validity among the three constructs (gender was excluded as a categorical demographic moderator). As shown in Table 2, the three-factor model demonstrated the best fit to the data: CMIN/DF = 1.902 (< 3), RMSEA = 0.063 (< 0.08), CFI = 0.93 (> 0.9), TLI = 0.922 (> 0.9), indicating good discriminant validity.

**Table 2:** Confirmatory factor analysis results.

Model	CMIN/DF	RMSEA	CFI	TLI
Three-factor	1.902	0.063	0.93	0.922
Two-factor	3.457	0.105	0.808	0.786
Single-factor	4.613	0.127	0.716	0.686

#### 4.3.3 Descriptive statistics and correlations

Descriptive statistics and correlations are presented in Table 3. Attitudes toward own aging were significantly negatively correlated with perceived occupational stress (*r* = −0.633, *p* < 0.01); perceived occupational stress was significantly negatively correlated with thriving at work (*r* = −0.623, *p* < 0.01); and attitudes toward own aging were significantly positively correlated with thriving at work (*r* = 0.495, *p* < 0.01). These results provide preliminary support for our theoretical model.

**Table 3:** Descriptive statistics and correlations.

Variables	*M*	SD	1	2	3	4	5	6	7	8
1. Age	48.35	2.878	–							
2. Education	4.75	0.818	−0.061	–						
3. Tenure	22.56	6.28	0.140^*^	−0.052	–					
4. Position level	2.46	0.79	0.038	0.212^**^	0.138^*^	–				
5. Gender	0.54	0.50	0.017	−0.010	0.002	0.057	–			
6. Attitudes toward own aging	3.398	1.09	0.139^*^	−0.004	0.136^*^	0.225^**^	−0.075	0.925		
7. Occupational stress	2.058	0.74	−0.079	0.056	0.005	−0.161^*^	−0.071	−0.633^**^	0.893	
8. Thriving at work	4.348	0.48	0.001	−0.092	−0.033	0.140^*^	0.057	0.495^**^	−0.623^**^	0.855

*N* = 225; *p < 0.05; **p < 0.01; ***p < 0.001.

#### 4.3.4 Hypothesis testing

We used SPSS 27.0 to test the main effects (Table 4). Model 5 showed that attitudes toward own aging had a significant positive effect on thriving at work (*β* = 0.22, *p* < 0.001), supporting H1. Model 2 showed that attitudes toward own aging had a significant negative effect on perceived occupational stress (*β* = −0.43, *p* < 0.001). Model 6, after introducing perceived occupational stress, showed that the effect of attitudes toward own aging on thriving remained significant (*β* = 0.08, *p* = 0.008), indicating partial mediation, supporting H2.

**Table 4:** Regression analysis results.

Variable	Occupational stress			Thriving at work		
	Model 1	Model 2	Model 3	Model 4	Model 5	Model 6
Constant	2.865	3.04	3.38	4.61	4.51	5.50
Age	−0.02	0.00	0.00	−0.00	−0.01	−0.01
Education	0.08	0.06	0.06	−0.07	−0.06	−0.04
Tenure	0.00	0.01	−0.01	−0.00	−0.01	−0.00
Position level	−0.17^**^	−0.04	−0.02	0.11	0.04	0.03
Gender			−0.17^*^			
Attitudes toward own aging		−0.43^***^	−0.53^***^		0.22^***^	0.08^**^
Occupational stress						−0.32^***^
Attitudes toward own aging× gender			0.21^**^			
*R* ^2^	0.04	0.41	0.447	0.04	0.27	0.42
Adjusted *R*^2^	0.02	0.40	0.43	0.02	0.25	0.40
*∆R* ^2^	0.02	0.38^***^	0.03^**^	0.02	0.23^***^	0.15^***^
*F*	2.35	31.04^***^	25.08^***^	2.23	2.84^***^	3.63^***^

Bootstrap analyses (5,000 resamples) further confirmed the mediating effect (see Table 5). The direct effect of attitudes toward own aging on thriving was 0.08, 95% CI [0.02, 0.14], excluding zero. The indirect effect via perceived occupational stress was 0.14, 95% CI [0.08, 0.21], excluding zero, again supporting H2.

**Table 5:** Bootstrap results for mediation.

Path	Effect	*SE*	95%CI	
			Lower	Upper
Indirect effect				
Attitudes toward own aging → occupational stress → thriving at work	0.14	0.03	0.08	0.21
Direct effect				
Attitudes toward own aging → thriving at work	0.08	0.03	0.02	0.14
Total effect	0.22	0.02	0.16	0.27

Model 3 in Table 4 shows that the interaction between attitudes toward own aging and gender was significantly related to perceived occupational stress (*β* = 0.21, *p* = 0.007), indicating that the negative relationship between positive attitudes toward own aging and perceived occupational stress was stronger for female employees, supporting H3. Simple slope analysis (see Figure 3) further revealed that for women, the negative slope was steeper [*β* = −0.59, *SE* = 0.05, *p* < 0.001, 95% CI (−0.70, −0.48)] than for men [*β* = −0.38, *SE* = 0.05, *p* < 0.001, 95% CI (−0.48, −0.28)].

**Figure 3:**
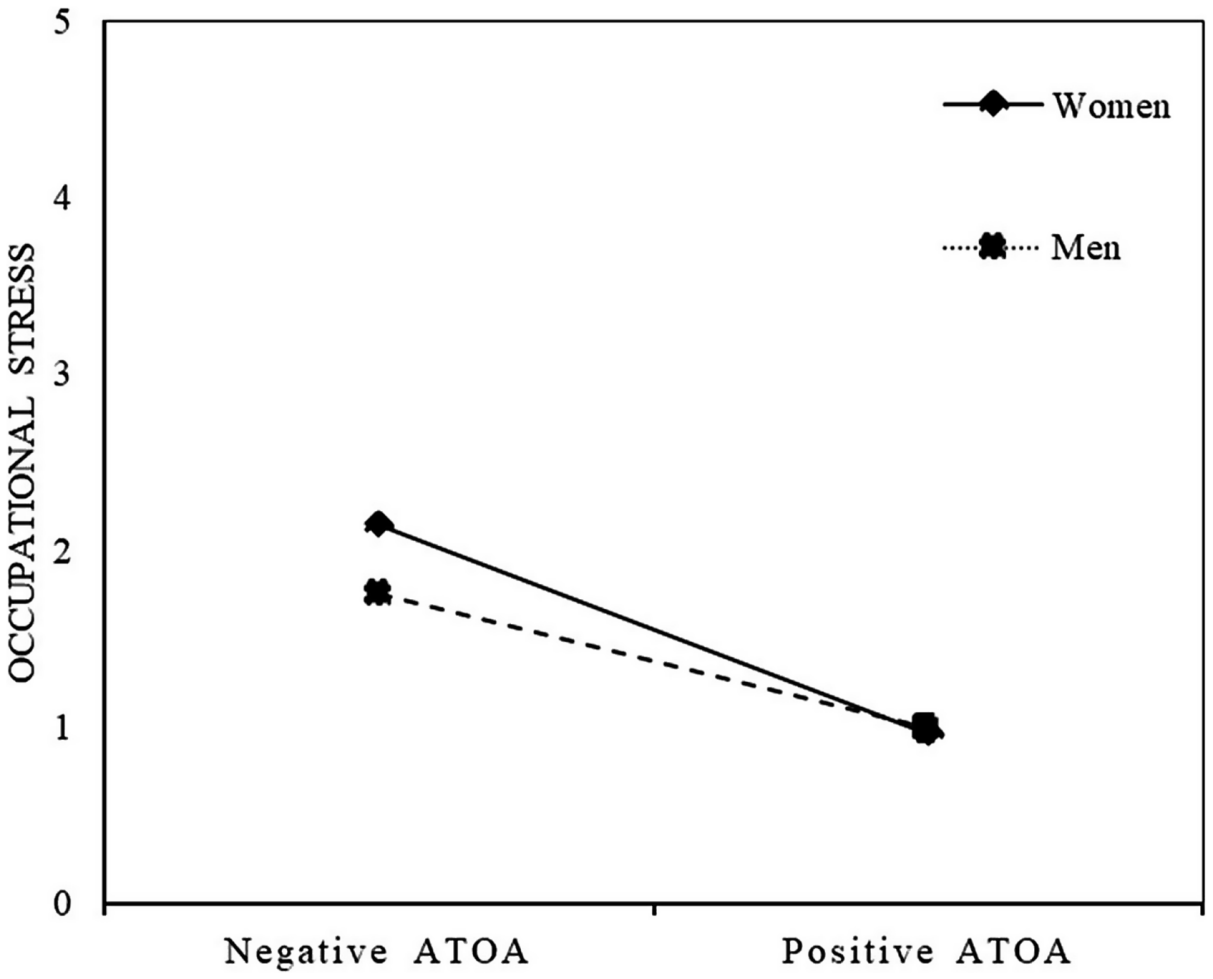
Moderating effect of gender (Study 2).

As shown in Table 6, the effect of moderated mediation was −0.06 (*SE* = 0.03), with a 95% CI [−0.14, −0.01], excluding zero, indicating that the moderated mediation effect was significant, and supporting H4.

**Table 6:** Moderated mediation results.

Moderator	Effect	*SE*	95%CI	
			Lower	Upper
Female	0.19	0.05	0.10	0.28
Male	0.12	0.03	0.06	0.19
Difference	−0.06	0.03	−0.14	−0.01

## 5 Discussion

### 5.1 Theoretical contributions

First, grounded in COR theory, this study focuses on attitudes toward own aging as a personal resource closely tied to workplace success, thereby extending research on aging attitudes from the fields of gerontology and geriatric medicine to the organizational management domain. Prior literature on attitudes toward own aging has largely focused on health outcomes and influencing factors among older adults (Benito-Rincón et al., 2026; Wettstein et al., 2020). The present study highlights the importance of conceptualizing attitudes toward own aging as a distinctive construct in the workplace. Although optimism, self-efficacy, positive affect, and future time perspective reliably predict individual work and health outcomes (Cao et al., 2024; Gong et al., 2026; Tao et al., 2025; Zhang and Huang, 2024), they do not capture individuals’ evaluations of own aging process. Attitudes toward own aging reflect the internalization of age stereotypes into the self-concept and represent a unique cognitive-evaluative process through which age-related beliefs become influential factors in workplace performance. By extending this construct to the organizational context, we demonstrate that employees’ evaluations of own aging process are not merely a health-related concern but a psychologically meaningful resource that shapes older employees’ work experiences. Older employees are undergoing a dual transition in both career and life, yet have received insufficient attention in previous research. This study not only extends COR theory but also validates that the value of personal resources lies not only in their positive attributes but in the thematic relevance between resources and the challenges individuals face. For example, when addressing the age discrimination and digital discrimination faced by older employees, it is important to recognize the value of older employees’ positive mindsets as a critical resource. Furthermore, this study challenges a deeply rooted assumption in everyday life: that older employees inevitably decline, lacking the vitality and learning capacity necessary for continued work contribution. By establishing the link between attitudes toward own aging and thriving at work, we demonstrate that chronological age does not predetermine older employees’ inability to achieve at work.

Second, this study identifies perceived occupational stress as a resource-depletion pathway that links attitudes toward own aging to the positive development of thriving at work, thereby enriching research on how aging-related self-evaluations influence employees’ work behaviors and states through resource dynamics. COR theory posits that stress arises from actual or anticipated resource loss (Hobfoll, 2001), yet prior research has rarely specified how aging-related cognitive appraisals trigger older employees’ perceptions of work resource loss. Positive attitudes toward own aging provide resources through a positive future time perspective and positive physiological and psychological cognitions (Kornadt et al., 2025), which help older employees attenuate occupational stress and reduce threat appraisals. The more negative older employees’ attitudes toward own aging, the more they lose confidence in controlling their lives and work, and thus fail to exhibit vitality at work (Benito-Rincón et al., 2026). By elucidating how aging self-attitudes facilitate resource reshaping to alleviate occupational stress and enhance thriving at work, this study underscores that resource appraisals are not solely determined by objective circumstances but are actively constructed through individuals’ domain-specific self-evaluations. Aging alters the subjective meaning of resource gain and loss—the same objective change (i.e., increasing age) may be appraised as either gain or loss depending on one’s aging attitudes. This study contributes to a more nuanced understanding of how resource dynamics operate across the career lifespan.

Third, this study reveals that gender differences lead to disparities in resource conversion, demonstrating that the resource-transfer pathway through which aging attitudes influence thriving via occupational stress varies across gender groups. Our findings shed light on the socially embedded nature of resource gain and loss processes, and confirm that women exhibit greater efficiency in converting positive aging cognitions into tangible resources. Men and women have long faced differentiated workplace resource constraints, and for older employees, these differences widen further with age. Therefore, we cannot ignore the differential efficacy of psychological resource utilization across genders (Putrevu, 2001). Moreover, these gender-based differences manifest not only in organizational settings but also in domestic spheres, where women bear a disproportionate share of caregiving responsibilities (Bianchi et al., 2000). Thus, in examining the relationship between aging attitudes and work behaviors, it is essential to account for the physiological and psychological distinctions between genders. Incorporating these factors into our research helps illuminate how the dynamics of work and family resources ultimately contribute to social harmony.

### 5.2 Practical implications

First, organizations should recognize the significant role of attitudes toward own aging in the workplace and cultivate positive attitudes among employees to enhance thriving. Enterprises can implement awareness campaigns and training programs that emphasize that age does not limit employee development, thereby reducing age discrimination and mitigating negative perceptions of aging. For example, organizations can regularly share success stories and growth experiences of mid-career backbone employees, highlighting their continuous learning, accumulated experience, and proactive approaches to solving company challenges, thereby helping employees realize that age brings gains in resources such as knowledge and experience. Enterprises may also design tailored career development paths for different age groups, alleviating concerns about career progression limitations associated with aging and providing diverse learning opportunities (e.g., online courses, internal training, industry seminars) to support continuous resource accumulation and a positive mindset toward aging. Furthermore, implementing age-inclusive HRM can effectively address age discrimination. Age-inclusive HRM emphasizes age diversity and equality, and through organizational culture, policies, and job design, it can systematically eliminate age-related biases and ensure equitable development opportunities for employees across all career stages (Boehm et al., 2014).

Second, organizations should emphasize workplace stress management and develop strategies for older employees. Specifically, enterprises can offer psychological counseling services to help employees manage stress. Workflow optimization can reduce the time and effort spent on repetitive tasks, allowing employees to focus on core business activities and thereby alleviating stress. By thoroughly reviewing existing workflows, organizations can identify cumbersome, repetitive, and inefficient steps and improve them by simplifying or automating them. Flexible work arrangements (e.g., flexible hours, remote work) and equitable access to training and development opportunities across all career stages also can further alleviate stress by accommodating life-stage circumstances and signaling that the organization values employees’ continued growth throughout their careers. Additionally, tasks should be assigned reasonably and flexibly based on employees’ capabilities, skills, and workloads to avoid overburdening or an imbalanced workload. Fair and objective performance appraisal systems should be established to accurately reflect employee performance and prevent undue stress caused by unreasonable evaluation criteria.

Third, organizations should adopt differentiated management approaches tailored to gender differences, paying particular attention to attitudes toward own aging and stress management among mid-career female employees (Man et al., 2026). For example, enterprises should ensure that female employees feel equally treated compared to male counterparts, alleviating their stress by breaking gender stereotypes and offering equal learning and promotion opportunities. Showcasing successful professional women within the organization can also be beneficial. Furthermore, task assignments should leverage the respective strengths of male and female employees, helping female employees recognize their own resource advantages and cultivate positive attitudes. Organizations should also examine whether their leaders and organizational culture genuinely support age and gender inclusion. For instance, by training managers to recognize age-related biases and ensuring transparency in promotion and development decisions. Ultimately, rather than focusing primarily on helping older employees adapt to aging, organizations should take responsibility for creating environments where employees can successful aging at work.

### 5.3 Limitations and future directions

First, this study employed experimental and survey methods with self-reported measures, which may introduce subjective bias and potentially affect the findings. Future research could adopt multi-source designs (e.g., combining self-ratings with supervisor or peer ratings), diary methods, and longitudinal designs, and supplement quantitative data with qualitative materials. Study 1 employed a scenario-based experimental design, which may have potential limitations regarding the manipulation. Although we designed the manipulation materials based on validated measurement scales, the manipulation may have inadvertently influenced participants’ mood or self-evaluations beyond the intended construct. Therefore, future research could refine the experimental manipulation through more precise designs to more clearly isolate the unique effects of attitudes toward own aging on workplace outcomes. For example, researchers could employ subtle priming techniques, such as using age-related words, to more specifically activate aging attitudes.

Second, this study adopted 45 years of age and above as the threshold for defining “older employees,” following established practices in prior research that sampled Chinese employees (Cui et al., 2025; Ma and Zhao, 2023). Nevertheless, we fully acknowledge that chronological definitions of “older employees” are inherently contested and context-dependent (Zacher, 2024), for example, some studies have employed 40 as the age cutoff (Liu et al., 2025). The experience and meaning of aging also vary considerably across industries and occupational contexts. In technology-intensive occupations, for instance, older workers face digital age discrimination, where the age threshold for being considered “old” can be as low as 35 (Shukis-Brown and Pritchard, 2026). This issue carries particular weight for the present investigation, as attitudes toward own aging may carry substantially different meanings across distinct life stages. Employees aged 45, 55, 65, and 75 are situated in fundamentally different career and life circumstances, with correspondingly divergent experiences of and responses to the aging process, and prior research has confirmed that chronological age is negatively associated with self-evaluations (Kornadt et al., 2018; Nagy et al., 2019a,b). Our further data analyses revealed that the negative relationship between positive aging attitudes and occupational stress was stronger in the younger age group [*β* = −0.55, *SE* = 0.04, 95% CI (−0.65, −0.46)] compared to the older age group [*β* = −0.32, *SE* = 0.06, 95% CI (−0.44, −0.20)], suggesting that the buffering effect of aging attitudes may diminish with advancing age (the simple slopes are presented in the Supplementary materials). Although we controlled for chronological age as a covariate and found our core results to remain robust, we recognize that treating age solely as a statistical control does not fully capture the nuanced developmental differences across the adult lifespan. Future research would benefit from employing more refined age categorizations, for instance, distinguishing between “Youthfuls,” “Matures,” and “Veterans” to examine whether the observed mechanisms operate differently across these subgroups within the older worker population (Nagy et al., 2019a,b). Adopting such a life-course perspective on workplace aging would more clearly illuminate how career stage and the transition toward retirement shape the actual utility of aging attitudes for older employees.

Third, although this study conceptualized attitudes toward own aging as a common antecedent of both learning and vitality, prior research suggests that these two dimensions may have distinct antecedents (Prem et al., 2017). Future research could conduct comparative analyses to disentangle the distinct pathways through which aging attitudes influence each dimension. Some scholars have called for multilevel conceptualizations of thriving beyond the individual level (Goh et al., 2022). Collective thriving, defined as the shared state of learning and vitality at the team, unit, or organizational level (Keister, 2014), represents collective growth and learning. Future research could adopt a team-level perspective to examine how attitudes toward own aging influence collective thriving, as well as the cross-level transmission of attitudes toward own aging across organizational levels. Furthermore, this study examined only one cognitive mechanism based on COR theory—perceived occupational stress. Other internal mechanisms and boundary conditions remain underexplored. For instance, drawing on Socioemotional Selectivity Theory, prior research indicates that aging attitudes and subjective age contribute to positive emotional experiences, yet the specific emotional responses of older employees have not been systematically examined (De Raedt et al., 2013). Future research could adopt alternative theoretical perspectives to further uncover these underlying mechanisms and enrich the literature.

Finally, this study examined the moderating role of gender but did not account for other factors that may differentiate aging attitudes and experiences between men and women. Existing research indicates that employees aged 45 and above often bear the dual burden of caring for aging parents and supporting grandchildren—responsibilities that substantially deplete family resources (Hou et al., 2025; Kossek et al., 2026). These caregiving demands may affect the robustness of our research model. Caregiving responsibilities are positively associated with work–family conflict and perceived stress, consuming personal resources such as time and energy (ten Brummelhuis and Bakker, 2012). Moreover, women continue to bear a disproportionate share of family care work (Bianchi et al., 2000). Therefore, in selecting gender as a moderator, future research should consider controlling for additional factors—such as the number of children and grandchildren, the number of elderly dependents, work–family conflict, caregiving intensity, and caregiving burden—to more precisely disentangle the effects of gender per se from those of caregiving responsibilities. Such investigations would provide a more complete picture of how family context shapes the resource dynamics underlying thriving at work among older employees. Furthermore, occupations characterized by high physical demands face more severe age-related challenges compared to those that value accumulated experience, professional expertise, or seniority (Maravelias, 2024). The present study did not collect detailed information on participants’ industry, occupation, or job characteristics. This represents an important limitation, as the meaning and experience of aging are likely to vary substantially across occupational contexts.

## 6 Conclusion

Drawing on one scenario-based experiment and one longitudinal questionnaire survey, this study tested the proposed model and found that positive attitudes toward own aging promote thriving at work among older employees. As a psychological resource, positive attitudes toward own aging reduce perceived occupational stress, which in turn enhances thriving at work. Perceived occupational stress mediates the relationship between attitudes toward own aging and thriving at work. Moreover, gender moderates this mediating effect, such that the indirect effect is stronger for older female employees than for older male employees.

## Data Availability

The original contributions presented in the study are included in the article/Supplementary material, further inquiries can be directed to the corresponding author.
