# Supplementary material for: Thriving despite aging: how attitudes toward own aging empower thriving at work
Source: Front Psychol. 2026 Sep 25;17:1959203. doi: 10.3389/fpsyg.2026.1959203 (PMC13649515; doi:10.3389/fpsyg.2026.1959203)
Supplement: Supplementary file 1 [file Table_3.DOCX]

Supplementary Material

## Supplementary Figures

**Supplementary Figure 1.** The figure is located in the 5.1Theoretical Contributions section, presenting the simple slopes for the moderating effect of age on the relationship between attitudes toward own aging and perceived occupational stress.
